# Competitive risk analysis of the therapeutic value of liver transplantation for liver cancer in children: A population-based study

**DOI:** 10.3389/fsurg.2022.938254

**Published:** 2022-08-31

**Authors:** Huiwu Xing, Chenyu Yang, Bingqian Tan, Mingman Zhang

**Affiliations:** Department of Hepatobiliary Surgery, Children's Hospital of Chongqing Medical University, Chongqing Key Laboratory of Pediatrics, National Clinical Research Center for Child Health and Disorders, Ministry of Education Key Laboratory of Child Development and Disorders, Chongqing, China

**Keywords:** liver transplantation, liver cancer, children, nomogram, prognosis

## Abstract

**Background:**

Liver transplantation (LT) is one of the most important treatments for children with liver cancer (CLCa) and has been increasingly used. However, there is a lack of large-scale and multicenter studies on the trend in the application and value of LT for the treatment of CLCa.

**Methods:**

We analyzed the clinicopathological data of CLCa from 2000 to 2018 from the Surveillance, Epidemiology, and End Results (SEER) database. We explored the trend in the application of LT for the treatment of CLCa. *LASSO Cox* regression and the *Log-Rank* test were used to explore prognostic factors, and we built a nomogram using the screened factors. Propensity score matching was used to balance the baseline data of patients undergoing LT and other surgeries, and then the *Log-Rank* test was used to evaluate the therapeutic value of LT for CLCa.

**Results:**

The 1-year, 3-year, 5-year, and 10-year overall survival (OS) rates of CLCa were 88.7%, 80.6%, 76.8%, and 73.0%, respectively. Then, we established a nomogram using many variables including age of diagnosis, regional lymph node metastasis, summary stage, and therapy. Internally validated and externally verified, our nomogram had good predictive power and clinical applicability. LT was increasingly being used to treat CLCa. There was no statistically significant difference in the OS of CLCa between the LT and other surgeries groups. After LT, the hepatoblastoma group had a better prognosis than the hepatocellular carcinoma group.

**Conclusion:**

We built a well-performing nomogram to predict the OS of CLCa. LT could improve the prognosis of CLCa as other surgeries and could be considered an effective treatment choice for CLCa.

## Introduction

Liver tumors in children are rare, but most of them (50%–60%) are malignant tumors (1, 2). Liver cancer accounts for approximately 1% of malignant tumors and 5%–6% of abdominal malignant tumors in children, including hepatoblastoma (HB), hepatocellular carcinoma (HCC), embryonal sarcoma, malignant rhabdoid tumor, hemangiosarcoma, and cholangiocarcinoma (1–3). Surgical resection is the first choice for the treatment of children with liver cancer (CLCa), but some patients may lose the opportunity for surgical resection due to anatomical or other reasons, and liver transplantation (LT) may be the only potential treatment choice (4). As one of the effective treatments for malignant liver tumors in adults, LT is also suitable for children (5–7) and has been used to treat CLCa for more than 50 years (8). In the United States, CLCa is one of the common indications for LT in children and is mainly used in the treatment of HB which is the most common liver cancer in children (6, 9, 10). With the application of chemotherapy and the progress of LT techniques, the prognosis of patients undergoing LT has been significantly improved (7, 10). However, the pathogenesis of liver cancers in children is related to congenital factors, embryonic development, and acquired mutagenesis, which is obviously different from that in adults. Therefore, even for the same kind of liver cancer, there are great differences in the occurrence, development, symptoms, treatment, and prognosis between children and adults.

Although CLCa has become one of the main indications for LT in children, there is a lack of large-scale cohort studies due to the small sample size. The SEER program currently covering 48.0% of the population of the United States could provide authoritative, multicenter, and long-term information on cancer statistics including cancer incidence and survival (https://seer.cancer.gov/). The SEER database has become one of the powerful tools to study epidemiology, therapeutic effect, and prognosis of various cancers (11–13).

In this study, we analyzed the application trend and efficacy of LT in the treatment of CLCa and discussed the factors affecting the curative effect. In addition, we also analyzed the prognostic factors of CLCa and further established a nomogram to evaluate the prognosis.

## Patients and methods

### Patients

**Figure 1:**
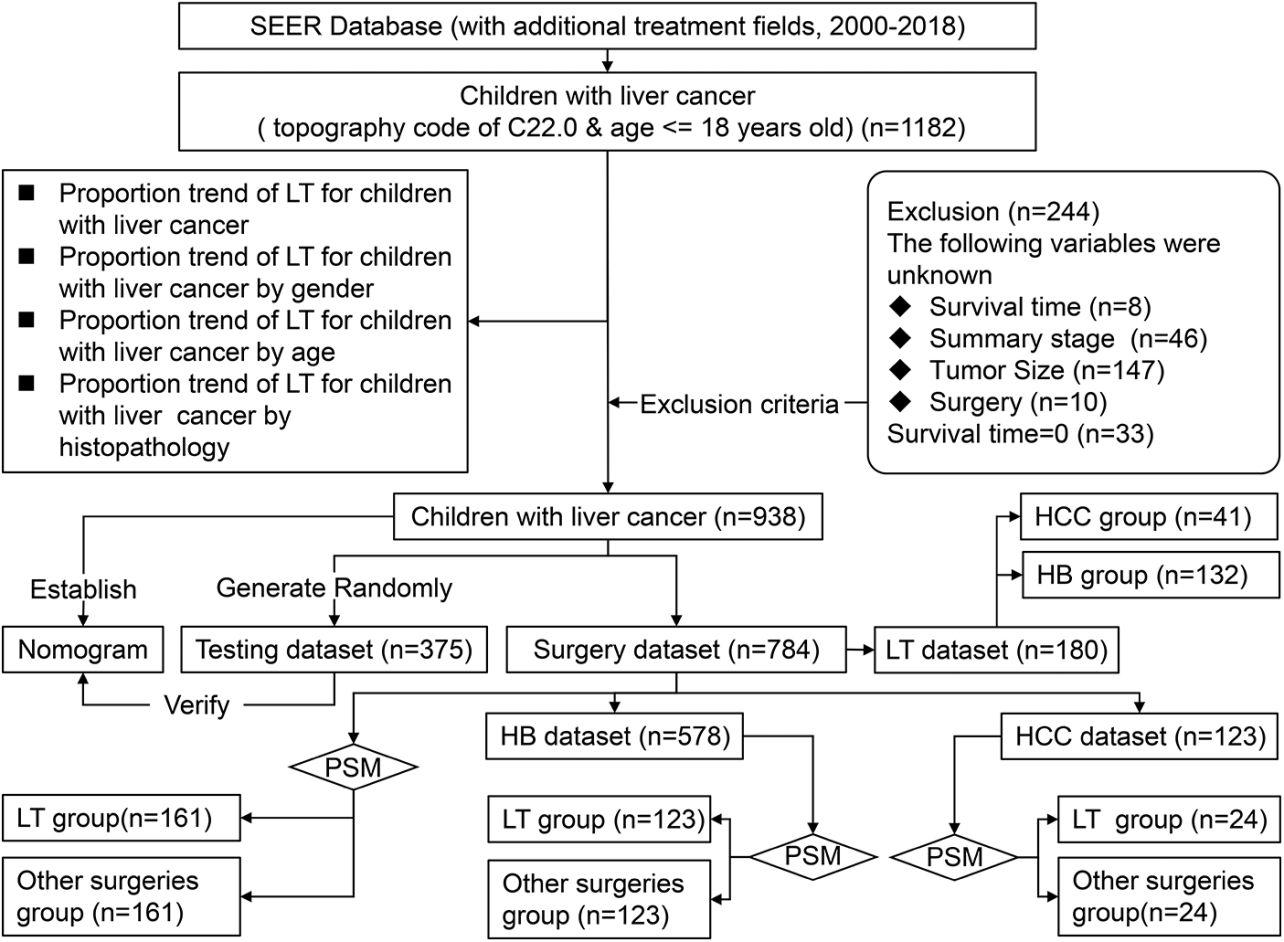
Flowchart of screening children with liver cancer in the SEER database.

We obtained demographic and clinicopathological data of CLCa from 2000 to 2018 online from the Case List Session of SEER*Stat software (version 8.3.9.2). Patients aged ≤18 years old with a site code of C22.0 were enrolled in this study (*n* = 1182). Patients with incomplete data or a survival time of less than 1 month were excluded from this study (Figure 1).

### Variable definition

Demographic data included age, gender, and race. Clinicopathological data included the year of diagnosis, alpha-fetoprotein (AFP), the American Joint Committee on Cancer (AJCC) staging system (8th edition), summary stage, histopathology, grade, and treatment options. Because the AJCC staging system (8th edition) was not used in the SEER database at the time of this study, the AJCC staging system (7th edition) was used in this study, including primary tumor (T), regional lymph node metastasis (N), and metastasis (M), which were used to evaluate HB and HCC in the SEER database. Because the AJCC prognostic stage groups do not apply to HB, the summary stage in SEER (including localized, regional, and distant) was used in this study (Figure 1). Age was classified into three groups: <2 years old, 2–6 years old, and 7–18 years old. Tumor size was classified into ≤50  mm and >50 mm. The absence of AFP in the HB group was too many, so AFP was not included in the analysis in the HB group. Overall survival (OS) in months was defined as the period from diagnosis to death due to any cause or the last follow-up.

### Study design

Before excluding patients according to the exclusion criteria, we analyzed the proportion of CLCa treated with LT and the trend of LT application. Because of the small sample size of CLCa, all the patients included in this study were not randomly divided into the training dataset and testing dataset as in other studies (14, 15). All the patients as the training dataset were used to establish a nomogram and 40% of them were randomly selected as the testing dataset to evaluate the effectiveness of the nomogram. We included patients who received local tumor destruction, segmental or wedge section, lobectomy, extended lobectomy, and LT as the surgery dataset and analyzed the factors affecting the prognosis of CLCa. Patients treated with surgery were divided into the LT group and the other surgeries group. Propensity score matching (PSM) was used to balance the baseline data between the LT group and the other surgeries group to reduce selection bias, and prognostic differences between groups were evaluated (Figure 1).

### Statistical analysis

The continuous variables were transformed into classification variables, which were shown as “frequency (percentage)”. The *Chi-square* test or *Fisher*'s exact test was used to compare the baseline data between groups. The *Kaplan-Meier* method was used to draw survival curves and calculate the 1-, 3-, 5-, and 10-year OS. The *Log-Rank* test was used to compare the difference in prognosis between groups. We performed penalized *Cox* regression using the adaptive least absolute shrinkage and selection operator (*LASSO*) to screen variables and improve overfitting and used the *Log-Rank* test to evaluate the relationship between screened variables and the prognosis of CLCa. The final screened variables were used to develop a nomogram. We used the area under the curve (AUC) of the receiver operating characteristic curve (ROC), the calibration plot, and the decision curve analysis (DCA) plot to evaluate the performance validity of the nomogram. Univariate and multivariate *Cox* regression analyses were used to screen the factors independently affecting the prognosis of CLCa undergoing LT. Before *LASSO Cox* regression and random split of the dataset, the seed number was set to 621 to ensure the repeatability of this study. A two-tailed *p* < .05 was considered statistically significant. All analyses were performed in *R* software (version 4.0.2) using *ggplot2*, *splines*, *rms*, *stringr*, *survival*, *survminer*, *tableone*, *caret*, *glmnet*, *timeROC*, *foreign*, *stdca.R*, *MatchIt*, and *optmatch* packages.

## Results

### Clinicopathological features

From 2000 to 2018, there were 1,182 CLCa in the SEER database. According to the exclusion criteria, 244 patients were excluded and 938 patients were included in this study (Figure 1). More patients were diagnosed from 2010 to 2018 than from 2000 to 2009 (54.1% vs. 45.9%), suggesting that the incidence of CLCa might increase. Most of the patients were male (59.2%), white (75.4%), had no lymph node metastasis (86.9%), had no distant metastasis (79.6%), and had HB (70.0%). More patients were less than 2 years old (48.6%) and had solitary localized tumors (T1, 42.3%; localized, 49.5%) (Table 1).

**Table 1:** Comparison of demographic and clinical characteristics between the overall and testing datasets.

	Overall (*n* = 938)	Testing dataset (*n* = 375)	*p-*value
Year of diagnosis			
2000–2009	431 (45.9%)	166 (44.3%)	0.623
2010–2018	507 (54.1%)	209 (55.7%)	
Gender			
Male	555 (59.2%)	224 (59.7%)	0.900
Female	383 (40.8%)	151 (40.3%)	
Age			
0–1 year old	456 (48.6%)	184 (49.1%)	0.680
2–6 years old	237 (25.3%)	101 (26.9%)	
7–18 years old	245 (26.1%)	90 (24.0%)	
Race			
White	707 (75.4%)	286 (76.3%)	0.927
Black	83 (8.8%)	33 (8.8%)	
Others	148 (15.8%)	56 (14.9%)	
AFP			
Negative	37 (3.9%)	17 (4.5%)	0.856
Positive	42 (4.5%)	18 (4.8%)	
Unknown	859 (91.6%)	340 (90.7%)	
Tumor size			
≤50 mm	133 (14.2%)	61 (16.3%)	0.381
>50 mm	805 (85.8%)	314 (83.7%)	
T			
T1	397 (42.3%)	166 (44.3%)	0.839
T2	137 (14.6%)	59 (15.7%)	
T3	215 (22.9%)	80 (21.3%)	
T4	81 (8.6%)	33 (8.8%)	
TX	108 (11.5%)	37 (9.9%)	
N			
N0	815 (86.9%)	326 (86.9%)	0.952
N1	57 (6.1%)	24 (6.4%)	
NX	66 (7.0%)	25 (6.7%)	
M			
M0	747 (79.6%)	308 (82.1%)	0.342
M1	191 (20.4%)	67 (17.9%)	
Summary stage			
Localized	464 (49.5%)	193 (51.5%)	0.558
Regional	282 (30.1%)	115 (30.7%)	
Distant	192 (20.5%)	67 (17.9%)	
Histopathology			
HCC	173 (18.4%)	71 (18.9%)	0.770
HB	657 (70.0%)	266 (70.9%)	
Others	108 (11.5%)	38 (10.1%)	
Grade			
Grade I	64 (6.8%)	26 (6.9%)	0.957
Grade II	49 (5.2%)	23 (6.1%)	
Grade III	27 (2.9%)	10 (2.7%)	
Grade IV	79 (8.4%)	34 (9.1%)	
Unknown	719 (76.7%)	282 (75.2%)	
Therapy			
None	16 (1.7%)	4 (1.1%)	0.447
Chemotherapy alone	138 (14.7%)	42 (11.2%)	
LT alone	27 (2.9%)	12 (3.2%)	
Other surgeries alone	74 (7.9%)	34 (9.1%)	
LT combined with chemotherapy	153 (16.3%)	71 (18.9%)	
Other surgeries combined with chemotherapy	530 (56.5%)	212 (56.5%)	

AFP, alpha-fetoprotein; LT, Liver transplantation; HB, hepatoblastoma; HCC, hepatocellular carcinoma.

### Nomogram construction and verification

**Figure 2:**
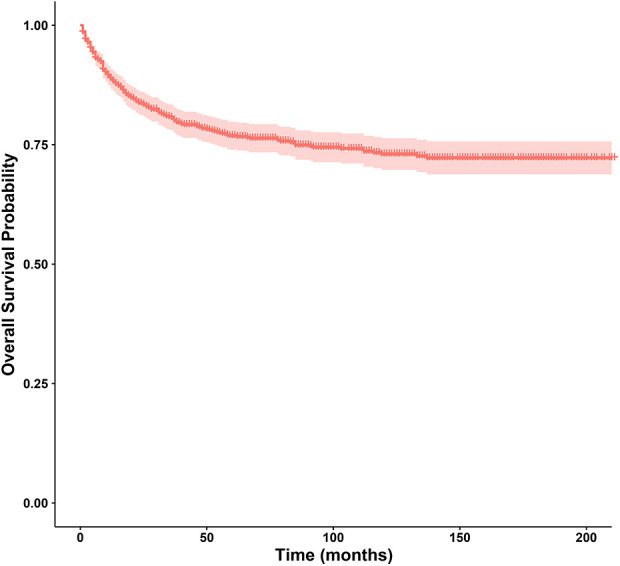
Survival curve of children with liver cancer.

**Figure 3:**
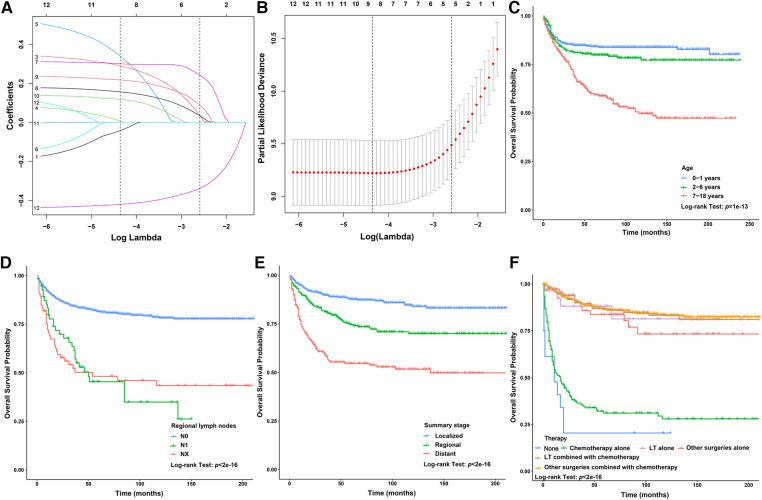
Screening variables closely related to the prognosis of children with liver cancer. (**A**) LASSO coefficient profiles of the variables of children with liver cancer. (**B**) Tenfold cross-validation for tuning parameter selection in the LASSO Cox model. (**C**) Survival curves of children with liver cancer between different age groups. (**D**) Survival curves of children with liver cancer between different N groups. (**E**) Survival curves of children with liver cancer between different summary stage groups. (**F**) Survival curves of children with liver cancer between different therapy groups.

**Figure 4:**
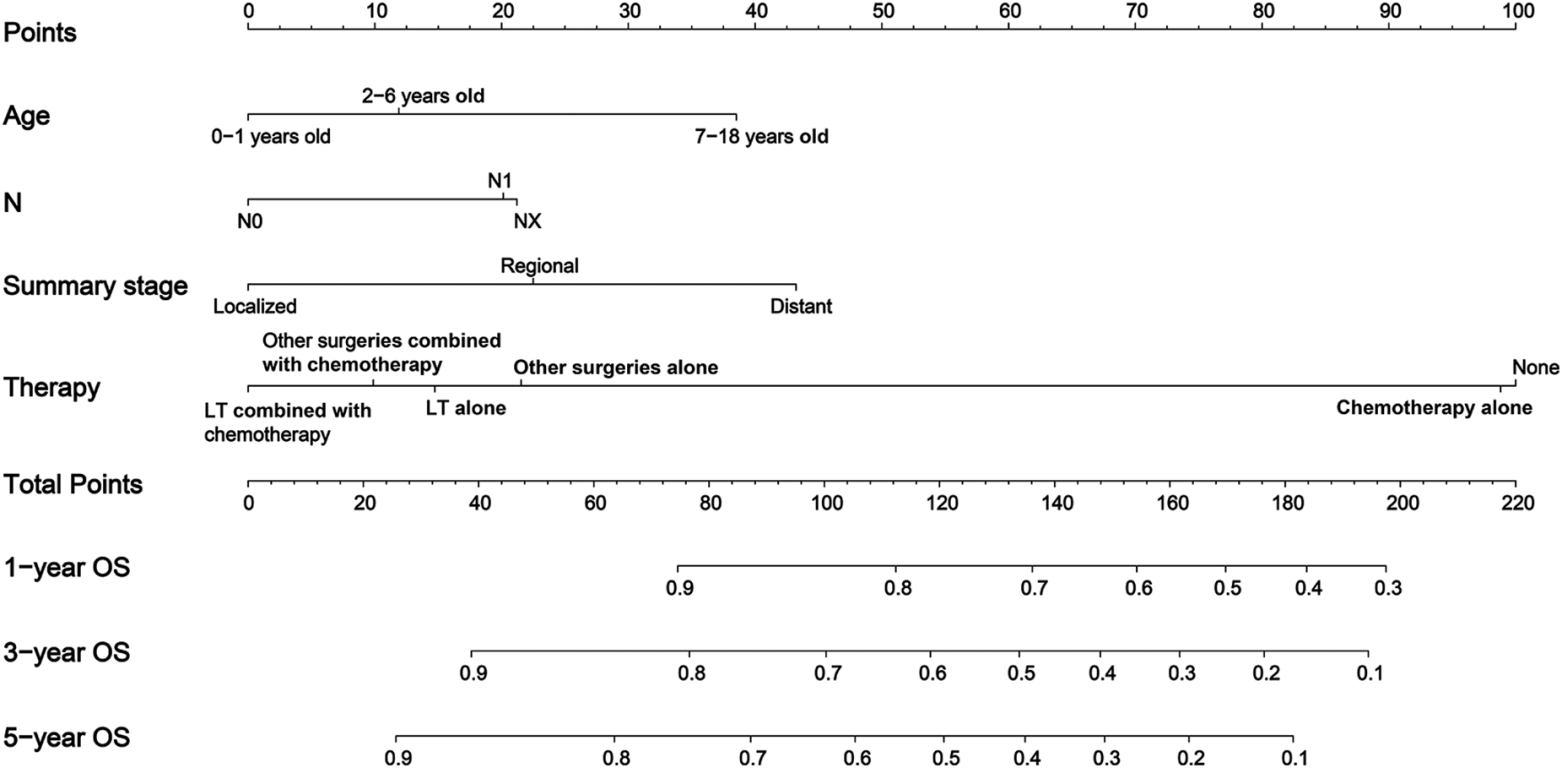
Nomogram for predicting the OS of children with liver cancer. OS, overall survival.

In this study, the 1-year, 3-year, 5-year, and 10-year OS rates of CLCa were 88.7%, 80.6%, 76.8%, and 73.0%, respectively (Figure 2). In the *LASSO Cox* regression, we use lambda (*λ*) as the penalty value to compress the coefficients of each variable, in which the later the coefficient of the variable is compressed to zero, the more important the variable is (Figure 3A); in addition, *λ* with as few variables as possible and an error as small as possible is considered the optimal penalty value (Figure 3B). We used *λ* of 1 time the standard error (se) (1 se *λ* = 0.081) as the penalty value to screen out four variables: age of diagnosis, N, summary stage, and therapy. The *Log-Rank* test results showed that the above four variables were closely related to the prognosis of CLCa (*p* < .05) (Figures 3C–F) and were used to construct a nomogram to predict the prognosis of CLCa (Figure 4).

**Figure 5:**
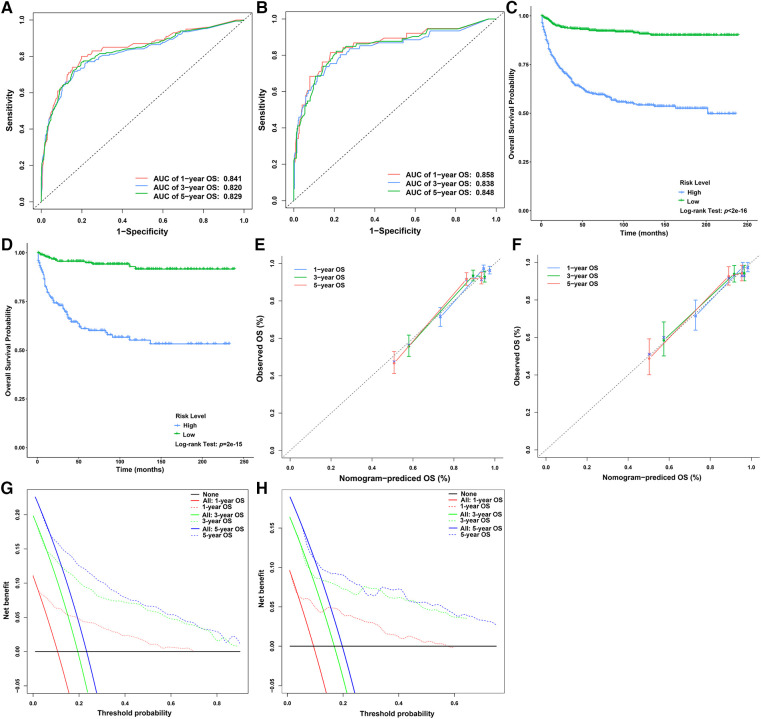
Verification of the nomogram for predicting the OS of children with liver cancer. (**A**) ROC curves of the 1-, 3-, and 5-year OS in the overall dataset. (**B**) ROC curves of the 1-, 3-, and 5-year OS in the testing dataset. (**C**) Survival curves of children with liver cancer between the high- and low-risk score groups in the overall dataset. (**D**) Survival curves of children with liver cancer between the high- and low-risk score groups in the testing dataset. (**E**) Calibration plots of the 1-, 3-, and 5-year OS in the overall dataset. (**F**) Calibration plots of the 1-, 3-, and 5-year OS in the testing dataset. (**G**) DCA plots of the 1-, 3-, and 5-year OS in the overall dataset. (**H**) DCA plots of the 1-, 3-, and 5-year OS in the testing dataset. DCA, decision curve analysis; ROC, receiver operating characteristic curve; OS, overall survival.

We used all the patients and the testing dataset to validate our prediction model internally and externally. There was no significant difference in baseline data between the training and testing datasets (Table 1). The AUCs of 1-year, 3-year, and 5-year OS predicted by all patients were 0.841, 0.820, and 0.829, respectively (Figure 5A), while the AUCs of 1-year, 3-year, and 5-year OS predicted by the testing dataset were 0.858, 0.838, and 0.848, respectively (Figure 5B). Patients were divided into high-risk-level and low-risk-level groups based on the risk score calculated according to our model. We found that the prognosis of the low-risk-level group was significantly better than that of the high-risk-level group (*p* < .05) (Figures 5C, D). Therefore, our model could well judge the prognosis of patients. Calibration plots using all patients and the testing dataset to predict 1-year, 3-year, and 5-year OS showed that the predicted results of the model were in good agreement with the ideal outcomes (Figures 5E, F). In addition, DCA plots showed that patients using our model to predict their prognosis could obtain a good net benefit, which suggested that our model had good clinical applicability (Figures 5G, H).

### Application trend of liver transplantation

**Figure 6:**
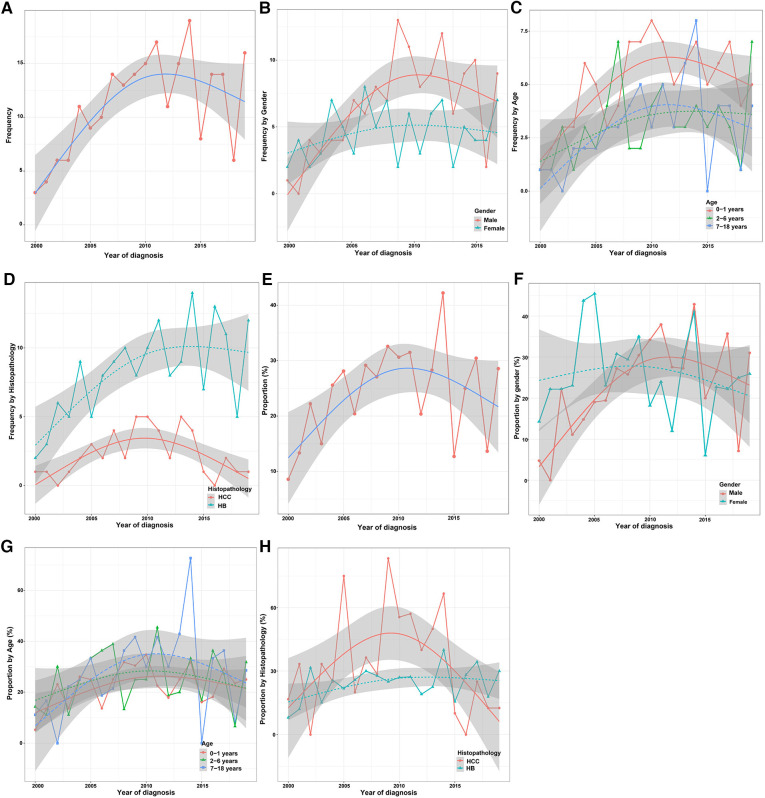
Trend in the application of LT for the treatment of children with liver cancer in different clinicopathological groups. (**A**) Trend in the application of LT for the treatment of children with liver cancer. (**B–D**) Trends in the application of LT for the treatment of children with liver cancer between different gender, age, and histopathology groups. (**E**) Proportion trend of LT for the treatment of children with liver cancer. (**F–H**) Proportion trends of LT for the treatment of children with liver cancer between different gender, age, and histopathology groups. LT, Liver transplantation.

Only a small number of CLCa received LT (19.2%) (Table 1), but LT was increasingly used in the treatment of CLCa (Figure 6A), and the proportion of LT in surgery had a similar trend (Figure 6E). The peak of LT application occurred in 2014 (19 cases, 39.6%) (Figures 6A, E). In addition, we found that more males, less than 2 years old, and HB patients received LT (Figures 6B–D). However, the proportion of LT in surgery tended to be similar in different gender, age, and histopathology groups (Figures 6F–H). Therefore, the application of LT in the treatment of CLCa has tended to increase, but growth has tended to be stable in recent years.

### Survival analysis

**Figure 7:**
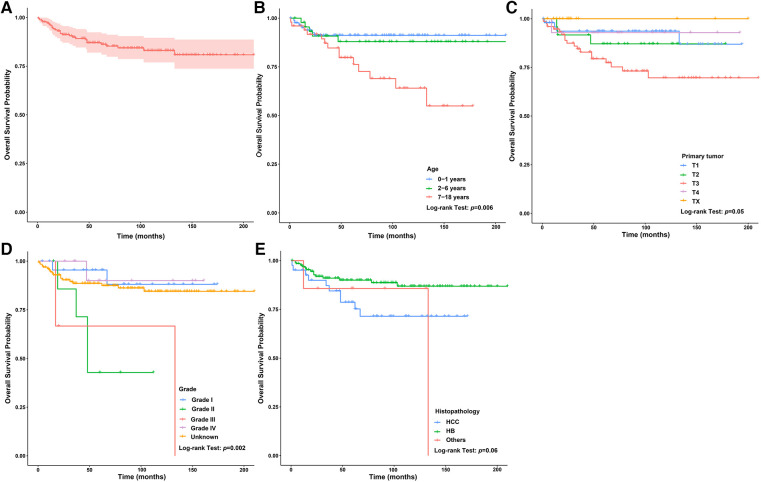
Survival curve of children with liver cancer treated with LT. (**A**) Survival curves of children with liver cancer treated with LT. (**B**) Survival curves of children with liver cancer treated with LT between different age of diagnosis groups. (**C**) Survival curves of children with liver cancer treated with LT between different T groups. (**D**) Survival curves of children with liver cancer treated with LT between different grade groups. (**E**) Survival curves of children with liver cancer treated with LT between different histopathology groups. LT, Liver transplantation.

In the surgery dataset, 180 patients underwent LT (180/784, 23.0%), of whom 154 survived (85.6%) and 26 died (14.4%) (Figure 1). The 1-year, 3-year, 5-year, and 10-year OS rates of CLCa undergoing LT were 96.0%, 89.9%, 87.0%, and 83.0%, respectively (Figure 7A). Univariate *Cox* regression analyses showed that age of diagnosis, T, grade, and histopathology were related to the prognosis of CLCa undergoing LT (Figures 7B–E and Table 2). Multivariate *Cox* regression analyses showed that T was an independent prognostic factor (Table 2).

**Table 2:** Univariate and multivariate regression analysis in the LT dataset.

	Univariate analysis		Multivariate analysis	
	HR (95% CI)	*p*-value	HR (95% CI)	*p*-value
Year of diagnosis				
2000–2009	1.000 [Reference]	–	/	/
2010–2018	0.916 [0.408, 2.055]	0.831	/	/
Gender				
Male	1.000 [Reference]	–	/	/
Female	0.957 [0.438, 2.090]	0.911	/	/
Age				
0–1 year old	1.000 [Reference]	–	1.000 [Reference]	–
2–6 years old	1.228 [0.389, 3.873]	0.726	1.114 [0.350, 3.540]	0.855
7–18 years old	3.551 [1.432, 8.807]	.006	1.806 [0.499, 6.541]	0.368
Race				
White	1.000 [Reference]	–	/	/
Black	1.812 [0.537, 6.110]	0.338	/	/
Others	0.779 [0.114, 2.079]	0.332	/	/
AFP				
Negative	1.000 [Reference]	–	/	/
Positive	0.544 [0.077, 3.868]	0.543	/	/
Unknown	0.405 [0.094, 1.736]	0.223	/	/
Tumor size				
≤50 mm	1.000 [Reference]	–	/	/
>50 mm	1.981 [0.594, 6.601]	0.266	/	/
T				
T1	1.000 [Reference]	–	1.000 [Reference]	–
T2	1.577 [0.352, 7.064]	0.551	1.983 [0.373, 10.530]	0.422
T3	3.315 [1.1120, 9.812]	.031	3.765 [1.034,13.709]	.044
T4	0.911 [0.102, 8.158]	0.934	1.158 [0.115, 11.653]	0.901
TX	6.7 × 10^−8^ [0.000, Inf]	0.997	6.0 × 10^−8^ [0.000, Inf]	0.998
N				
N0	1.000 [Reference]	–	/	/
N1	1.335 [0.313, 5.697]	0.696	/	/
NX	2.435 [0.724, 8.189]	0.150	/	/
M				
M0	1.000 [Reference]	–	/	/
M1	0.810 [0.279, 2.352]	0.698	/	/
Stage				
Localized	1.000 [Reference]	–	/	/
Regional	1.908 [0.704, 5.174]	0.204	/	/
Distant	1.280 [0.343, 4.771]	0.713	/	/
Grade				
Grade I	1.000 [Reference]	–	1.000 [Reference]	–
Grade II	6.698 [1.221, 36.742]	.029	3.406 [0.602, 19.258]	0.166
Grade III	10.220 [1.428, 73.150]	.021	5.735 [0.447, 73.553]	0.180
Grade IV	0.890 [0.081, 9.824]	0.924	0.448 [0.023, 8.564]	0.594
Unknown	1.563 [0.361, 6.767]	0.551	2.249 [0.426, 11.874]	0.340
Histopathology				
HCC	1.000 [Reference]	–	1.000 [Reference]	–
HB	0.420 [0.187, 0.947]	.037	0.629 [0.161, 2.449]	0.503
Others	1.188 [0.260, 5.421]	0.825	2.653 [0.217, 32.462]	0.445
Chemotherapy				
None	1.000 [Reference]	–	/	/
Chemotherapy	0.873 [0.300, 2.538]	0.803	/	/

AFP, alpha-fetoprotein; LT, Liver transplantation; HB, hepatoblastoma; HCC, hepatocellular carcinoma; HR, hazard ratio; CI, confidence interval.

### Therapeutic value of liver transplantation

**Figure 8:**
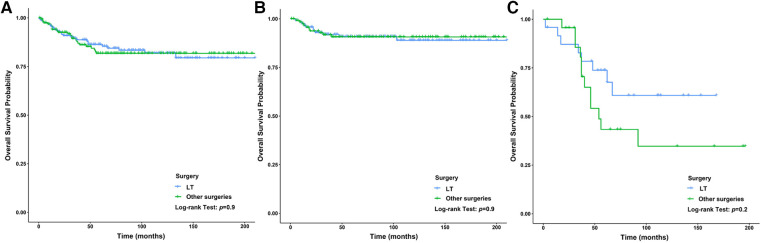
Survival curves of children with liver cancer between the LT group and the other surgeries group. (**A**) Survival curves of children with liver cancer after PSM between different therapy groups. (**B**) Survival curves of children with HB after PSM between different therapy groups. (**C**): Survival curves of children with HCC after PSM between different therapy groups. LT, Liver transplantation; HB, hepatoblastoma; HCC, hepatocellular carcinoma; PSM, propensity score matching.

We analyzed the relationship between the method of operation and the prognosis of CLCa but found that the difference in baseline data between the LT group and the other surgeries group was statistically significant, so PSM was used to balance the baseline data between the two groups (Supplementary Table S1). After PSM, we found that there was no significant difference in OS between the two groups (*p* > .05) (Figure 8A). We used the PSM method to balance the difference in the baseline data between the LT group and other surgeries group in children with HB (*p* > .05) (Supplementary Table S2) and found that the prognosis of the LT group was similar to that of the other surgeries group in children with HB (*p* > .05) (Figure 8B). Similar results were also found in children with HCC (*p* > .05) (Supplementary Table S3 and Figure 8C), but LT appeared to improve the long-term prognosis of children with HCC (Figure 8C). In addition, we analyzed the relationship between histopathology and the prognosis of CLCa treated with LT and found that the prognosis of children with HCC was significantly worse than that of patients with HB (*p* < .05) (Figure 8E and Supplementary Figure S1). The 5-year OS rates of HCC and HB groups were 76.8% and 90.0%, respectively (Figure 8E and Supplementary Figure S1).

## Discussion

In this population-based retrospective study, we found that the prognosis of CLCa was good with 5-year and 10-year OS rates of 76.8% and 73.0%, respectively, and we also constructed a nomogram with good performance to predict OS. LT has been increasingly widely used in the treatment of CLCa and could improve the prognosis of CLCa, with 5-year and 10-year OS rates of 87.0% and 83.0%, respectively, especially in children with HCC. In addition, although there were more children with HB receiving LT than children with HCC receiving LT, the proportions of LT among the two groups were similar. We found that age of diagnosis, T, grade, and histopathology were associated with the prognosis of CLCa undergoing LT. After PSM, we found that LT could be considered an effective choice for the treatment of CLCa.

Although the incidence of CLCa is low, CLCa is one of the most common abdominal malignant tumors in children and is increasing yearly (2, 16). A study based on the SEER database showed that the 5-year OS rates of HB and HCC were 52.4% and 18.0%, respectively, from 1979 to 1996 (2). Another study showed that the 5-year OS of CLCa was 68.5% from 1985 to 2013 in the United States (17). OS rates of CLCa in this study were higher than those in the above studies, which may suggest that the prognosis of CLCa has improved recently. Studies have reported that the prognosis of CLCa was closely related to age, year of diagnosis, stage, histopathology type, and therapy (17, 18). We performed the penalization procedure using *LASSO Cox* regression to address overfitting and miscalibration, which has been widely used in medical research (19–21). In this study, we found that age of diagnosis, N, summary stage, and therapy were included and closely related to the prognosis of CLCa and built a nomogram using the above factors to predict the OS of CLCa. We found that age of 0–1 years old, no lymph node metastasis, localized stage, and comprehensive therapy were prognostic factors for longer OS. Comprehensive therapy could be more beneficial to improving the prognosis of CLCa than a single treatment, and LT might be more beneficial to improving the prognosis of CLCa than other surgeries. Through internal and external verification, our predictive model performed well in judging the prognosis of CLCa.

LT could completely remove the tumor focus, similar to total hepatectomy, and solve the problem of insufficient liver function reserve. LT combined with chemotherapy could improve the prognosis of patients and expand the scope of indications for LT (7, 22–24). LT has become an important method for the treatment of CLCa (6). In this study, we found that LT has been increasingly used in the treatment of CLCa in all gender, age, and histopathology groups, but the growth trends have plateaued in recent years. We found that the proportions and change trends of LT were similar in all gender, age, and histopathology groups, which was similar to the results of other studies (7). Therefore, LT has been an important treatment choice for CLCa, similar to other surgeries (10).

In this study, the prognosis of CLCa treated with LT was good, with 1-year, 3-year, 5-year, and 10-year OS rates of 96.0%, 89.9%, 87.0%, and 83.0%, respectively. Studies have shown that LT could be considered an effective option for the treatment of CLCa to improve prognosis, especially when complete tumor excision is unlikely to be performed by partial hepatectomy (10, 25). Studies have shown that tumor burden including tumor size and tumor number, biomarkers including AFP and NLR, post-treatment extent of disease (POST-TEXT) stages, tumor differentiation, vascular invasion, and chemotherapy could predict the prognosis of liver cancer patients undergoing LT (25, 26). We found that age of diagnosis, T, grade, and histopathology were closely related to the prognosis of CLCa treated with LT, especially T. In our nomogram, we found that CLCa undergoing LT may have a better prognosis than CLCa undergoing other surgeries. We found that the OS rate of the LT group was similar to that of the other surgeries group, in all patients, the HCC group, and the HB group. However, children with HCC undergoing LT seemed to have a better long-term prognosis than those undergoing other surgeries. After LT, the prognosis of children with HB was significantly better than that of children with HCC, which was in accordance with the results of other studies (10, 27, 28). However, we cannot ignore the fact that the prognosis of children with HB is better than that of children with HCC (2, 17).

Our study still has several limitations. First, data in the SEER database are not always complete, and some patients were excluded because of the lack of data, which may cause bias in the inclusion of the patients and selection of variables. Second, this study is retrospective, which may also lead to selection bias. In addition, limited by the source of data, we could not explore the role of many variables in the prognosis of CLCa undergoing LT, such as POST-TEXT stages, complications, and chemotherapy. Nevertheless, our study adds new information to understand the application and value of LT in the treatment of CLCa.

## Conclusion

In conclusion, LT has been increasingly used to treat CLCa and could improve their prognosis. The prognosis of CLCa treated with LT could be similar to that of CLCa treated with other surgeries, in relation to the age of diagnosis, T, grade, and histopathology. We established a well-performing nomogram using the age of diagnosis, N, summary stage, and therapy to predict the OS of CLCa.

## Data Availability

The original contributions presented in the study are included in the article/**Supplementary Material**, further inquiries can be directed to the corresponding author/s.
