# Supplementary material for: Competitive risk analysis of the therapeutic value of liver transplantation for liver cancer in children: A population-based study
Source: Front Surg. 2022 Aug 31;9:938254. doi: 10.3389/fsurg.2022.938254 (PMC9470878; doi:10.3389/fsurg.2022.938254)
Supplement: Supplementary file 1 [file Table_1_v1.docx]

**Supplementary table 1. Comparison of demographic, clinical characteristics of the surgery dataset before and after PSM**

|  | **before PSM (180 vs. 604)** | | | **after PSM (161 vs. 161)** | | |
| --- | --- | --- | --- | --- | --- | --- |
|  | **LT** | **Other surgeries** | ***p*** | **LT** | **Other surgeries** | ***p*** |
| **Year of Diagnosis** | | | | | | |
| 2000-2009 | 76 (42.2%) | 272 (45.0%) | 0.561 | 68 (42.2%) | 76 (47.2%) | 0.433 |
| 2010-2018 | 104 (57.8%) | 332 (55.0%) |  | 93 (57.8%) | 85 (52.8%) |  |
| **Gender** |  | | | | | |
| Male | 104 (57.8%) | 357 (59.1%) | 0.817 | 92 (57.1%) | 97 (60.2%) | 0.651 |
| Female | 76 (42.2%) | 247 (40.9%) |  | 69 (42.9%) | 64 (39.8%) |  |
| **Age** | | | | | | |
| 0-1 years old | 84 (46.7%) | 306 (50.7%) | 0.319 | 77 (47.8%) | 84 (52.2%) | 0.734 |
| 2-6 years old | 46 (25.6%) | 163 (27.0%) |  | 43 (26.7%) | 40 (24.8%) |  |
| 7-18 years old | 50 (27.8%) | 135 (22.4%) |  | 41 (25.5%) | 37 (23.0%) |  |
| **Race** | | | | | | |
| White | 138 (76.7%) | 459 (76.0%) | 0.914 | 123 (76.4%) | 129 (80.1%) | 0.508 |
| Black | 14 (7.8%) | 53 (8.8%) |  | 13 (8.1%) | 8 (5.0%) |  |
| Others | 28 (15.6%) | 92 (15.2%) |  | 25 (15.5%) | 24 (14.9%) |  |
| **Tumor Size** | | | | | | |
| <=50mm | 37 (20.6%) | 79 (13.1%) | 0.018 | 19 (11.8%) | 17 (10.6%) | 0.860 |
| >50mm | 143 (79.4%) | 525 (86.9%) |  | 142 (88.2%) | 144 (89.4%) |  |
| **T** | | | | | | |
| T1 | 50 (27.8%) | 307 (50.8%) | <0.001 | 42 (26.1%) | 56 (34.8%) | 0.155 |
| T2 | 25 (13.9%) | 94 (15.6%) |  | 20 (12.4%) | 16 (9.9%) |  |
| T3 | 75 (41.7%) | 91 (15.1%) |  | 71 (44.1%) | 52 (32.3%) |  |
| T4 | 14 (7.8%) | 48 (7.9%) |  | 13 (8.1%) | 18 (11.2%) |  |
| TX | 16 (8.9%) | 64 (10.6%) |  | 15 (9.3%) | 19 (11.8%) |  |
| **N** | | | | | | |
| N0 | 160 (88.9%) | 547 (90.6%) | 0.76 | 142 (88.2%) | 140 (87.0%) | 0.898 |
| N1 | 11 (6.1%) | 29 (4.8%) |  | 10 (6.2%) | 10 (6.2%) |  |
| NX | 9 (5.0%) | 28 (4.6%) |  | 9 (5.6%) | 11 (6.8%) |  |
| **M** | | | | | | |
| M0 | 148 (82.2%) | 513 (84.9%) | 0.447 | 134 (83.2%) | 128 (79.5%) | 0.474 |
| M1 | 32 (17.8%) | 91 (15.1%) |  | 27 (16.8%) | 33 (20.5%) |  |
| **Stage** | | | | | | |
| Localized | 53 (29.4%) | 371 (61.4%) | <0.001 | 44 (27.3%) | 49 (30.4%) | 0.453 |
| Regional | 95 (52.8%) | 142 (23.5%) |  | 90 (55.9%) | 79 (49.1%) |  |
| Distant | 32 (17.8%) | 91 (15.1%) |  | 27 (16.8%) | 33 (20.5%) |  |
| **Histopathology** | | | | | | |
| HCC | 41 (22.8%) | 82 (13.6%) | <0.001 | 27 (16.8%) | 30 (18.6%) | 0.637 |
| HB | 132 (73.3%) | 446 (73.8%) |  | 128 (79.5%) | 122 (75.8%) |  |
| Others | 7 (3.9%) | 76 (12.6%) |  | 6 (3.7%) | 9 (5.6%) |  |
| **Grade** | | | | | | |
| Grade I | 24 (13.3%) | 31 (5.1%) | 0.003 | 12 (7.5%) | 12 (7.5%) | 0.218 |
| Grade II | 9 (5.0%) | 30 (5.0%) |  | 8 (5.0%) | 15 (9.3%) |  |
| Grade III | 3 (1.7%) | 21 (3.5%) |  | 2 (1.2%) | 7 (4.3%) |  |
| Grade IV | 13 (7.2%) | 59 (9.8%) |  | 10 (6.2%) | 7 (4.3%) |  |
| Unknown | 131 (72.8%) | 463 (76.7%) |  | 129 (80.1%) | 120 (74.5%) |  |
| **Chemotherapy** | | | | | | |
| None | 27 (15.0%) | 74 (12.3%) | 0.401 | 14 (8.7%) | 25 (15.5%) | 0.088 |
| Chemotherapy | 153 (85.0%) | 530 (87.7%) |  | 147 (91.3%) | 136 (84.5%) |  |
