# Supplementary material for: Competitive risk analysis of the therapeutic value of liver transplantation for liver cancer in children: A population-based study
Source: Front Surg. 2022 Aug 31;9:938254. doi: 10.3389/fsurg.2022.938254 (PMC9470878; doi:10.3389/fsurg.2022.938254)
Supplement: Supplementary file 2 [file Table_2_v1.docx]

**Supplementary table 2. Comparison of demographic and clinical characteristics of the HB group before and after PSM**

|  | **before PSM (446 vs. 132)** | | | **after PSM (123 vs. 123)** | | |
| --- | --- | --- | --- | --- | --- | --- |
|  | **LT** | **Other surgeries** | ***p*** | **LT** | **Other surgeries** | ***p*** |
| **Year of Diagnosis** | | | | | | |
| 2000-2009 | 53 (40.2%) | 194 (43.5%) | 0.560 | 46 (37.4%) | 56 (45.5%) | 0.244 |
| 2010-2018 | 79 (59.8%) | 252 (56.5%) |  | 77 (62.6%) | 67 (54.5%) |  |
| **Gender** |  | | | | | |
| Male | 82 (62.1%) | 270 (60.5%) | 0.821 | 77 (62.6%) | 75 (61.0%) | 0.896 |
| Female | 50 (37.9%) | 176 (39.5%) |  | 46 (37.4%) | 48 (39.0%) |  |
| **Age** | | | | | | |
| 0-1 years old | 75 (56.8%) | 295 (66.1%) | 0.004 | 71 (57.7%) | 86 (69.9%) | 0.091 |
| 2-6 years old | 43 (32.6%) | 135 (30.3%) |  | 41 (33.3%) | 32 (26.0%) |  |
| 7-18 years old | 14 (10.6%) | 16 (3.6%) |  | 11 (8.9%) | 5 (4.1%) |  |
| **Race** | | | | | | |
| White | 103 (78.0%) | 339 (76.0%) | 0.716 | 95 (77.2%) | 96 (78.0%) | 0.745 |
| Black | 12 (9.1%) | 37 (8.3%) |  | 11 (8.9%) | 8 (6.5%) |  |
| Others | 17 (12.9%) | 70 (15.7%) |  | 17 (13.8%) | 19 (15.4%) |  |
| **Tumor Size** | | | | | | |
| <=50mm | 17 (12.9%) | 63 (14.1%) | 0.825 | 15 (12.2%) | 12 (9.8%) | 0.683 |
| >50mm | 115 (87.1%) | 383 (85.9%) |  | 108 (87.8%) | 111 (90.2%) |  |
| **T** | | | | | | |
| T1 | 35 (26.5%) | 228 (51.1%) | <0.001 | 35 (28.5%) | 34 (27.6%) | 1 |
| T2 | 12 (9.1%) | 68 (15.2%) |  | 12 (9.8%) | 12 (9.8%) |  |
| T3 | 57 (43.2%) | 66 (14.8%) |  | 50 (40.7%) | 50 (40.7%) |  |
| T4 | 13 (9.8%) | 31 (7.0%) |  | 12 (9.8%) | 12 (9.8%) |  |
| TX | 15 (11.4%) | 53 (11.9%) |  | 14 (11.4%) | 15 (12.2%) |  |
| **N** | | | | | | |
| N0 | 115 (87.1%) | 423 (94.8%) | <0.001 | 115 (93.5%) | 117 (95.1%) | 0.485 |
| N1 | 8 (6.1%) | 3 (0.7%) |  | 5 (4.1%) | 2 (1.6%) |  |
| NX | 9 (6.8%) | 20 (4.5%) |  | 3 (2.4%) | 4 (3.3%) |  |
| **M** | | | | | | |
| M0 | 108 (81.8%) | 383 (85.9%) | 0.314 | 102 (82.9%) | 101 (82.1%) | 1 |
| M1 | 24 (18.2%) | 63 (14.1%) |  | 21 (17.1%) | 22 (17.9%) |  |
| **Stage** | | | | | | |
| Localized | 35 (26.5%) | 284 (63.7%) | <0.001 | 34 (27.6%) | 36 (29.3%) | 0.929 |
| Regional | 73 (55.3%) | 99 (22.2%) |  | 68 (55.3%) | 65 (52.8%) |  |
| Distant | 24 (18.2%) | 63 (14.1%) |  | 21 (17.1%) | 22 (17.9%) |  |
| **Grade** | | | | | | |
| Grade I | 4 (3.0%) | 20 (4.5%) | 0.101 | 3 (2.4%) | 6 (4.9%) | 0.198 |
| Grade II | 2 (1.5%) | 2 (0.4%) |  | 1 (0.8%) | 1 (0.8%) |  |
| Grade III | 0 (0.0%) | 8 (1.8%) |  | 0 (0.0%) | 3 (2.4%) |  |
| Grade IV | 6 (4.5%) | 8 (1.8%) |  | 6 (4.9%) | 2 (1.6%) |  |
| Unknown | 120 (90.9%) | 408 (91.5%) |  | 113 (91.9%) | 111 (90.2%) |  |
| **Chemotherapy** | | | | | | |
| None | 2 (1.5%) | 26 (5.8%) | 0.072 | 2 (1.6%) | 4 (3.3%) | 0.679 |
| Chemotherapy | 130 (98.5%) | 420 (94.2%) |  | 121 (98.4%) | 119 (96.7%) |  |
