# Supplementary material for: Competitive risk analysis of the therapeutic value of liver transplantation for liver cancer in children: A population-based study
Source: Front Surg. 2022 Aug 31;9:938254. doi: 10.3389/fsurg.2022.938254 (PMC9470878; doi:10.3389/fsurg.2022.938254)
Supplement: Supplementary file 3 [file Table_3_v1.docx]

**Supplementary table 3. Comparison of demographic and clinical characteristics of the HCC group before and after PSM**

|  | **before PSM (82 vs. 41)** | | | **after PSM (24 vs. 24)** | | |
| --- | --- | --- | --- | --- | --- | --- |
|  | **LT** | **Other surgeries** | ***p*** | **LT** | **Other surgeries** | ***p*** |
| **Year of Diagnosis** | | | | | | |
| 2000-2009 | 20 (48.8%) | 41 (50.0%) | 1 | 12 (50.0%) | 13 (54.2%) | 1 |
| 2010-2018 | 21 (51.2%) | 41 (50.0%) |  | 12 (50.0%) | 11 (45.8%) |  |
| **Gender** |  | | | | | |
| Male | 21 (51.2%) | 48 (58.5%) | 0.563 | 10 (41.7%) | 15 (62.5%) | 0.248 |
| Female | 20 (48.8%) | 34 (41.5%) |  | 14 (58.3%) | 9 (37.5%) |  |
| **Age** | | | | | | |
| 0-1 years old | 8 (19.5%) | 2 (2.4%) | 0.004 | 0 (0.0%) | 0 (0.0%) | 1 |
| 2-6 years old | 3 (7.3%) | 5 (6.1%) |  | 2 (8.3%) | 1 (4.2%) |  |
| 7-18 years old | 30 (73.2%) | 75 (91.5%) |  | 22 (91.7%) | 23 (95.8%) |  |
| **Race** | | | | | | |
| White | 28 (68.3%) | 64 (78.0%) | 0.058 | 16 (66.7%) | 17 (70.8%) | 0.065 |
| Black | 2 (4.9%) | 9 (11.0%) |  | 1 (4.2%) | 5 (20.8%) |  |
| Others | 11 (26.8%) | 9 (11.0%) |  | 7 (29.2%) | 2 (8.3%) |  |
| **AFP** | | | | | | |
| Negative | 6 (14.6%) | 24 (29.3%) | 0.024 | 4 (16.7%) | 7 (29.2%) | 0.441 |
| Positive | 14 (34.1%) | 12 (14.6%) |  | 7 (29.2%) | 4 (16.7%) |  |
| Unknown | 21 (51.2%) | 46 (56.1%) |  | 13 (54.2%) | 13 (54.2%) |  |
| **Tumor Size** | | | | | | |
| <=50mm | 19 (46.3%) | 13 (15.9%) | 0.001 | 7 (29.2%) | 7 (29.2%) | 1 |
| >50mm | 22 (53.7%) | 69 (84.1%) |  | 17 (70.8%) | 17 (70.8%) |  |
| **T** | | | | | | |
| T1 | 12 (29.3%) | 36 (43.9%) | 0.076 | 8 (33.3%) | 8 (33.3%) | 0.767 |
| T2 | 11 (26.8%) | 20 (24.4%) |  | 4 (16.7%) | 3 (12.5%) |  |
| T3 | 17 (41.5%) | 17 (20.7%) |  | 12 (50.0%) | 12 (50.0%) |  |
| T4 | 1 (2.4%) | 6 (7.3%) |  | 0 (0.0%) | 0 (0.0%) |  |
| TX | 0 (0.0%) | 3 (3.7%) |  | 0 (0.0%) | 1 (4.2%) |  |
| **N** | | | | | | |
| N0 | 38 (92.7%) | 60 (73.2%) | 0.037 | 22 (91.7%) | 16 (66.7%) | 0.084 |
| N1 | 3 (7.3%) | 20 (24.4%) |  | 2 (8.3%) | 6 (25.0%) |  |
| NX | 0 (0.0%) | 2 (2.4%) |  | 0 (0.0%) | 2 (8.3%) |  |
| **M** | | | | | | |
| M0 | 36 (87.8%) | 69 (84.1%) | 0.787 | 21 (87.5%) | 21 (87.5%) | 1 |
| M1 | 5 (12.2%) | 13 (15.9%) |  | 3 (12.5%) | 3 (12.5%) |  |
| **Stage** | | | | | | |
| Localized | 16 (39.0%) | 43 (52.4%) | 0.182 | 10 (41.7%) | 10 (41.7%) | 1 |
| Regional | 20 (48.8%) | 26 (31.7%) |  | 11 (45.8%) | 11 (45.8%) |  |
| Distant | 5 (12.2%) | 13 (15.9%) |  | 3 (12.5%) | 3 (12.5%) |  |
| **Grade** | | | | | | |
| Grade I | 20 (48.8%) | 11 (13.4%) | <0.001 | 11 (45.8%) | 3 (12.5%) | 0.082 |
| Grade II | 7 (17.1%) | 26 (31.7%) |  | 4 (16.7%) | 6 (25.0%) |  |
| Grade III | 2 (4.9%) | 8 (9.8%) |  | 1 (4.2%) | 3 (12.5%) |  |
| Grade IV | 2 (4.9%) | 0 (0.0%) |  | 1 (4.2%) | 0 (0.0%) |  |
| Unknown | 10 (24.4%) | 37 (45.1%) |  | 7 (29.2%) | 12 (50.0%) |  |
| **Chemotherapy** | | | | | | |
| None | 25 (61.0%) | 44 (53.7%) | 0.563 | 15 (62.5%) | 13 (54.2%) | 0.77 |
| Chemotherapy | 16 (39.0%) | 38 (46.3%) |  | 9 (37.5%) | 11 (45.8%) |  |
