# Supplementary figures and images for: Competitive risk analysis of the therapeutic value of liver transplantation for liver cancer in children: A population-based study
Source: Front Surg. 2022 Aug 31;9:938254. doi: 10.3389/fsurg.2022.938254 (PMC9470878; doi:10.3389/fsurg.2022.938254)

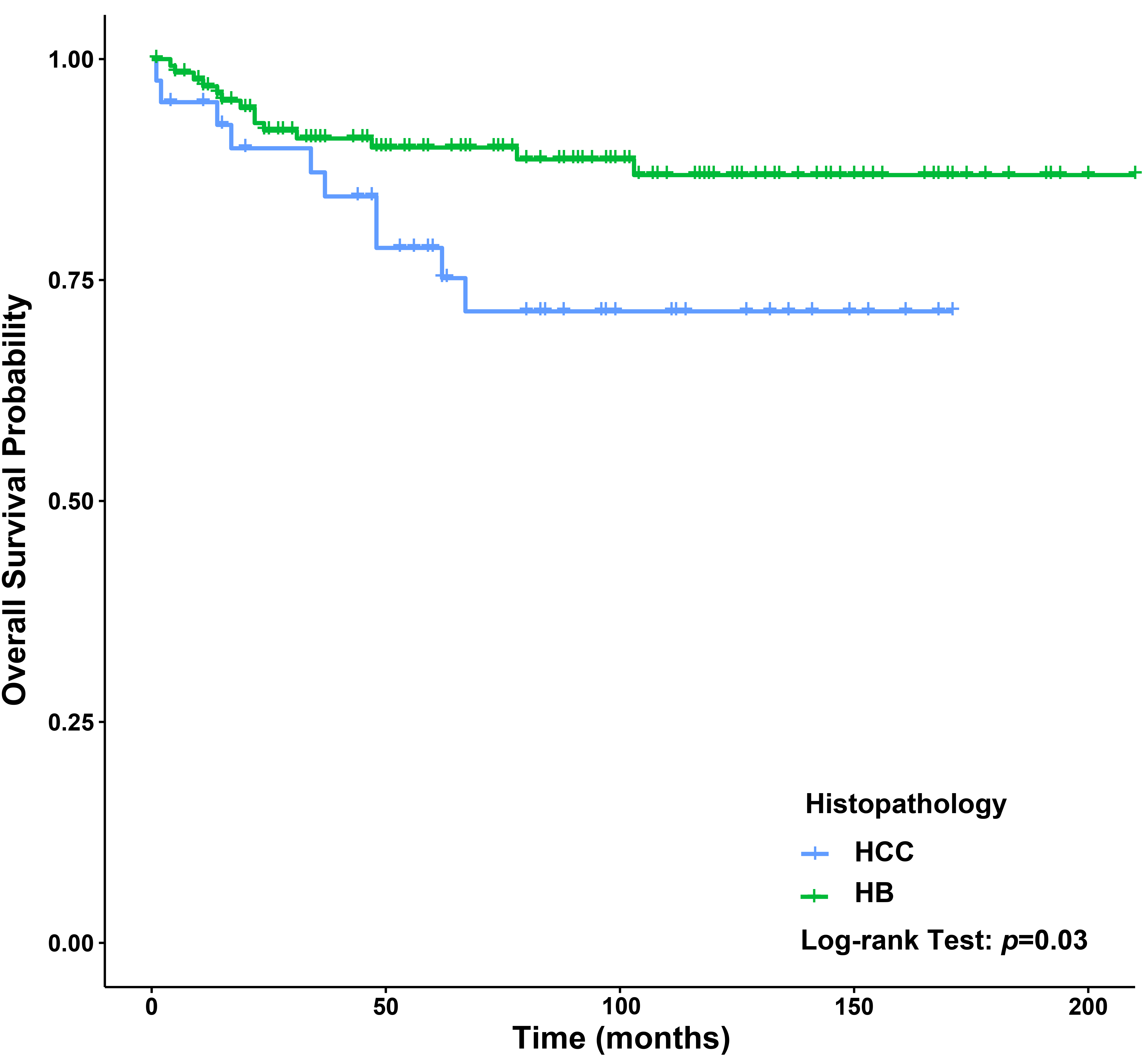

Supplement: Supplementary file 4 [file Image_1_v1.tif]
